# Target-Specificity in Scorpions; Comparing Lethality of Scorpion Venoms across Arthropods and Vertebrates

**DOI:** 10.3390/toxins9100312

**Published:** 2017-10-04

**Authors:** Arie van der Meijden, Bjørn Koch, Tom van der Valk, Leidy J. Vargas-Muñoz, Sebastian Estrada-Gómez

**Affiliations:** 1 CIBIO Research Centre in Biodiversity and Genetic Resources, InBIO, Rua Padre Armando Quintas 7, 4485-661 Vairão, Vila do Conde, Portugal; 2 Institute of Biology Leiden, Leiden University, P.O. Box 9505, 2300 RA Leiden, The Netherlands; b.e.v.koch@biology.leidenuniv.nl (B.K.); Tom.vandervalk@ebc.uu.se (T.v.d.V.); 3 Facultad de Medicina, Universidad Cooperativa de Colombia, Calle 50 A No 41-20, Medellín 050012, Antioquia, Colombia; leidy.vargasmu@campusucc.edu.co; 4 Programa de Ofidismo/Escorpionismo—Serpentario, Universidad de Antioquia UdeA, Carrera 53 No 61-30, Medellín 050010, Antioquia, Colombia; sestradas@gmail.com

**Keywords:** scorpions, venom, LD_50_, zebrafish, chicken, mealworm, waxworm

## Abstract

Scorpions use their venom in defensive situations as well as for subduing prey. Since some species of scorpion use their venom more in defensive situations than others, this may have led to selection for differences in effectiveness in defensive situations. Here, we compared the LD_50_ of the venom of 10 species of scorpions on five different species of target organisms; two insects and three vertebrates. We found little correlation between the target species in the efficacy of the different scorpion venoms. Only the two insects showed a positive correlation, indicating that they responded similarly to the panel of scorpion venoms. We discuss the lack of positive correlation between the vertebrate target species in the light of their evolution and development. When comparing the responses of the target systems to individual scorpion venoms pairwise, we found that closely related scorpion species tend to elicit a similar response pattern across the target species. This was further reflected in a significant phylogenetic signal across the scorpion phylogeny for the LD_50_ in mice and in zebrafish. We also provide the first mouse LD_50_ value for *Grosphus grandidieri*.

## 1. Introduction

Venom peptides are adapted to their role in altering the target organism’s physiology [1]. The high level of biological activity in venoms evolves in an arms race with a specific class of target organisms [2,3], and is impacted by the range of target organisms [4]. Whereas snakes will mostly prey upon as well as need to defend themselves from vertebrates, scorpions use their venom to incapacitate their prey, which consists primarily of arthropods, but also use the same venom to defend against predators, which often include vertebrates. Therefore, scorpion venoms faced evolutionary pressure to be effective in both arthropods and vertebrates simultaneously. Although scorpions rarely feed on vertebrates, several vertebrate specific [5,6] and arthropod-specific [7,8,9] venom compounds have been identified in scorpion venoms [5]. Scorpions therefore could be involved in evolutionary arms races against their predators, as well as against their prey. The intensity of these races may be different, depending on the ecological circumstances and speed of diversification [10]. Since prey may lose their life in an encounter, while predators only risk to lose their meal, selection for traits that may lead to a positive outcome of an encounter is usually stronger in prey than in predators (the “life-dinner principle”, [11]). However, when prey are dangerous, this dynamic could be reversed [12]. It has been argued that predator venom and prey defenses evolve rapidly in an arms race, while defensive venom compounds primarily cause pain, and evolve less quickly [13]. Pain, however, may not be the only mediator of defensive effect. 

Defensive use of venom can be beneficial to scorpions by promoting immediate deterrence, learned avoidance, and/or innate avoidance in the predator. Immediate deterrence, for instance by causing immediate pain or other noxious effects to the attacker, is effective but requires close contact with the attacker in every encounter, as by definition venom needs to be injected into the body of the attacker, carrying a significant risk of injury or death to the defending venomous animal. Learned avoidance, which may follow a successful deterrence event, provides the benefit that the individual predator will avoid the species in future encounters, thus reducing the further necessity of injecting venom to attain the deterrent effect, and thus avoiding potential harm. However, other individuals of the same predator species may not have learned this behavior, even if some amount cultural transmission could exist [14]. Encounters with individuals of the predator species that have not learned to avoid scorpions would lead to more encounters that could endanger the scorpion. An entire predator species can also evolve an innate avoidance behavior. In this case, all members of the species would avoid eating a particular prey species. For instance, South American birds that prey on small snakes display an innate avoidance of coral snakes [15,16] leading to Batesian mimicry of the color pattern by harmless snakes [17]. For such avoidance behaviors to evolve in a predator species, an encounter with a venomous prey animal must be detrimental to fitness. In this case, pain alone may not be sufficient, and partial incapacitation or death would be a much stronger selective pressure [12]. Pain may therefore not be the only, or even most important, mediator of the defensive capacity of venom in some systems. Scorpions are recognized by predators as potentially harmful [18,19], suggesting innate avoidance and therefore strong selection on predator avoidance. Several cases of potential Batesian mimicry of scorpion models exist, such as geckoes [20,21] and solifuges [22]. The capacity of scorpions to harm or kill their predators in order to elicit such innate avoidance may therefore also be a significant selective pressure on the side of the scorpions, and this could be a significant factor in the evolution of venom compounds with a high lethality to vertebrates. 

One of the basic metrics of lethality of venom is the LD_50_, or the dose at which half the tested population dies. The LD_50_ is a simple test to gauge venom efficacy in a certain class of target organisms, and is important in the development of antisera [23]. However, this metric is rather limited in its relevance to ecological function, as for prey incapacitation, immobilization may be more important than mortality [24]. It also does not take into account the deterrent effect of pain caused to predators. In addition, even a species with highly potent venom may simply not carry sufficient venom to cause harm to a larger predator, or may behaviorally meter its venom to rarely inject large amounts of it [25,26]. Venom amount must therefore also be taken into account when assessing the ecological role of venom.

LD_50_ tests are traditionally conducted on laboratory mice, under the assumption that the mouse is a good model for other vertebrates. First, mouse data are often construed as representative for venom efficacy in humans, ignoring the differences in responses to toxic stimuli between mouse and human [27]. Other organisms have also been used for LD_50_ assays, such as chicken embryos [28,29,30], blowflies [24] and several other insects [31]. The latter study showed large differences between the responses of different target species to spider venom.

Scorpions differ highly in their defensive use of venom [32]; buthid scorpions generally rely on their stinger in defensive situations, whereas representatives of other families, particularly the Scorpionidae, rely more on their powerful chelae. This could suggest that scorpion venoms are diverse in their efficacy against predator and prey. We here compared the LD_50_ of the venom of several buthid and non-buthid species in arthropods and vertebrates. We expect species that are in an arms race with vertebrate predators to show high toxicity in vertebrates compared to arthropods, whereas species that primarily use venom in prey incapacitation are expected to show a high toxicity to arthropods. 

## 2. Results

The range of LD_50_ values for all insect species is at most an order of magnitude, while for the vertebrates, the LD_50_ ranges over two orders of magnitude (Table 1). T-tests showed that there are no statistically significant differences between buthids and non-buthids in either arthropod LD_50_, but did show a significant difference between these two groups for *Danio* (*p* = 7.72 × 10^−5^), and to a lesser extent for *Mus* (*p* = 1.86 × 10^−3^), with the buthids having a higher toxicity to *Mus*, and a lower toxicity to *Danio*. This was also evident from the significant phylogenetic signal in the LD_50_ of *Danio* and *Mus* across the scorpion tree (Table 2). There was no phylogenetic signal detected across the scorpion species in their effect across the target organism tree (data not shown). 

The results of the correlations (Table 3) show that several scorpion species show similar response patterns over the five target species; *Pandinus*, *Heterometrus*, *Iurus*, and *Hadrurus* appear to provoke a similar response pattern across the target species, with relatively low LD_50_ values in *Galleria*, *Danio*, and *Gallus*, but relatively high values in *Tenebrio* and *Mus*. High correlation coefficients were also found between members of the family Buthidae, but these were not significant after correction for multiple comparisons. An exception was the buthid *Buthus*, which did not show significant correlation with any other species. *Hadrurus* and *Iurus* showed a high correlation. These representatives of the families Caraboctonidae and Iuridae respectively, were considered taxonomically closely related [43], but are now considered to be phylogenetically more distant [44]. The same pattern, in which the Buthidae and non-Buthidae appear to have the highest correlations amongst themselves held when venom volume (Table S2 in Supplementary Materials) was factorized with the LD_50_. 

There was no correlation between the target species (Table 4). Only *Galleria* and *Tenebrio* show a similar response curve to our panel of 10 scorpion venoms. However, this result was not borne out by the Spearman rank correlation. *Danio* and *Mus* show a significant negative correlation, indicating an opposite response to the venom panel. 

We observed several toxicological symptoms of the venom of *Grosphus grandidieri* in mice. These symptoms appeared at different times in every group after the venom injection. Table S3 in the supplementary materials shows all symptoms detected per group.

## 3. Discussion

The buthid scorpions in this study showed a higher toxicity to *Mus*, but the pattern in *Gallus* and *Danio* was the opposite. This may be because buthid scorpions specifically target rodents, and have been in an arms race with certain rodents in their environment. *Mus* may not have evolved or secondarily lost any evolved resistance against scorpion venom, and could therefore be particularly susceptible to buthid venom. On the other hand, the difference may also be the result of testing *Danio* and *Gallus* in the embryonal stage. These embryos may simply be less susceptible to buthid venoms because they may express fewer or different specific targets for the venom compounds to interact with, such as ion channels. If this is the case, the LD_50_ values ascertained using embryo assays may not be representative of the toxic effects in adults of these target species. The reverse signal in *Danio* is of interest as it seems to indicate that, if buthid neurotoxins have less effect on these embryos, the non-buthids have evolved toxic compounds that buthids do not possess or do not express in significant amounts. However, if the stark differences between *Danio*, *Gallus*, and *Mus* are not due to the developmental stage, they may be indicative of a high level of specialization in targeting of scorpion venoms. Our results clearly show that the vertebrate assay systems used here cannot be used interchangeably, and each is a poor indicator for lethality in the other. In fact, differences in susceptibility to scorpion venom between different vertebrate species have long been known from non-quantitative data [45]. Our data suggests that there may be no feasible universal vertebrate system to test the lethality of different scorpion venoms. Students of the ecological importance of scorpion venom may need to study the effect of venom on each target group separately. 

We assumed that scorpions rarely include vertebrates in their diet, and that vertebrate toxicity can therefore be an indicator for the use of scorpion venom in defense against predators. However, some arthropods do regularly prey on scorpions, and vertebrates are by no means the only, or in some cases possibly not even the main predators on scorpions. In particular cases, a low arthropod LD_50_ could have evolved due to predatory pressure by other arthropods on scorpions, including other scorpions [46,47]. Adding other potential predator or prey groups, such as arachnids, myriapods, crustaceans, reptiles, and amphibians would be of interest. However, inclusion of several scorpion species from different parts of the world, both for buthids and non-buthids, should negate the effect of particular local predator–prey relationships. We found no phylogenetic signal in the LD_50_ values of the arthropod species. Also, the *t*-tests showed no significant difference between buthid and non-buthid species in arthropod LD_50_. It seems therefore that arthropod toxicity is less variable among scorpion species. This may be due to arthropods expressing only a single variant of voltage gated sodium channel [48,49]. Alternatively, since the differences between *Gallus*, *Danio*, and *Mus* may be the result of the former two being tested in an early stage of development, the large difference in variability between the arthropod LD_50_ values and the *Mus* LD_50_ values could be indicative of a selective pressure on scorpion venom for high lethality to vertebrate predators. Since the family Buthidae is considered monophyletic, this difference may be the result of a single evolutionary event.

## 4. Materials and Methods

### 4.1. Species Selection

We selected 10 scorpion species from three families in order to sample a broad range of venom potencies. We selected five target species consisting of two arthropods (the larvae of the wax moth, *Galleria mellonella*; and mealworms, *Tenebrio molitor*) and three vertebrates (zebrafish embryos, *Danio rerio*; chicken embryos, *Gallus gallus*; and mice, *Mus musculus*). Ethical approval for the mouse experiments was granted by the Comité de Ética para la Experimentación con Animales—Universidad de Antioquia on August 2015, document 98.

### 4.2. Venom Preparation

Venom was extracted from live adult or sub-adult scorpions by applying a voltage to the metasoma, alternating between 0 and 18 V, at a rate of 45 Hz and a duty factor of 10%. The contact points were wetted with saline to facilitate electrical conductance. The telson was not part of the circuit, thereby avoiding any changes to the venom due to the applied voltage. Venom was collected in low protein-binding 2 mL tubes (Simport, Beloeil, QC, Canada), frozen in liquid nitrogen, and stored at −20 °C until they were lyophilized. The amount of dry venom content per specimen was obtained by dividing the total dry mass by the number of extractions required to obtain it. Scorpions were given a resting period of at least two weeks between consecutive milkings. Unless otherwise stated, venom preparations were made by dissolving lyophilized venom in Hank’s balanced salt solution, and vortexing or shaking with glass beads at 30 Hz until homogeneously suspended. 

### 4.3. In Vivo Assays

Five types of in vivo assays were performed:

(1) *Chicken Assay*. Venom solutions were applied to three days old chicken embryos, and mortality was ascertained after 24 h by candling the eggs. See [30] for details on this method.
(2) *Zebrafish Assay*. WT (ABTL) zebrafish embryos were injected with serially diluted venom solutions at three days post fertilization (DPF) in sample sizes of 20 embryos per venom concentration. Embryos were anesthetized with 200 μg/mL buffered 3-aminobenzoic acid (Tricaine, Sigma-Aldrich, St. Louis, MO, USA), in eggwater (60 μg/mL Instant Ocean Sea Salt, (Spectrum Brands, Blacksburg, VA, USA)), and venom solutions were delivered intravenously in 5 nL volume by injection into the Duct of Cuvier as previously described [50]. Survival was monitored at 24 h post injection by visual inspection of heartbeat. Embryonic bodyweight was estimated as the average drained weight of 30 embryos at three DPF, measured in triplicate on a high precision scale.
(3) *Mealworm Assay*. Every mealworm was individually weighed on a high precision scale. Venom solutions were applied to mealworms of 127.8 ± 14.1 mg body mass by using a 10 µL Hamilton syringe (Hamilton, Reno, NV, USA). Venom was injected laterally on the ventral side, between the sixth and seventh abdominal segment, keeping the needle as close to the body wall as possible to avoid damaging the internal organs. Different dosages of venoms were tested; 0.125, 0.25, 0.5, 1, and 2 µg/mg bodyweight, with 12 individuals per treatment. As control, individuals were injected with Hank’s balanced salt solution, (*n* = 12 per venom treatment). Higher concentrations for *Babycurus jacksoni* and *Buthus ibericus* where needed to calculate an accurate LD_50_ and therefore additional treatments of 4 and 2.8 µg/mg bodyweight respectively was performed. Mortality was assessed over a five-day period by looking at color change (mealworms turn black quickly after death) and by applying physical stimuli to elicit a response.
(4) *Waxworm Assay*. The waxworm assay was similar to the mealworm assay. Waxworms at the last instar before pupation were used. Different dosages of venoms were tested, 0.1, 0.25, 0.5, and 1 µg/mg bodyweight, with 12 individuals per treatment. As control, individuals were injected with Hank’s balanced salt solution (*n* = 12 per venom treatment). Additional treatments for *Centruroides gracilis* and *Androctonus australis* were needed to accurately calculate LD_50_, 2 and 0.025 µg/mg bodyweight respectively. Mortality was assessed over a five-day period by looking at color change (waxworms turn brown/black quickly after death) and by applying physical stimuli to elicit a response.
(5) *Mouse Assay*. The LD_50_ test was carried out on male albino Swiss mice of approximately 19 g body weight. Different amounts of venom from *G. grandidieri* were tested in parallel; 5.2 mg/kg, 21.1 mg/kg and 50 mg/kg (group 1, group 2, and group 3 respectively). Three mice were used in each dose and in the negative control. Injections were performed intraperitoneally using physiological saline solution as vehicle and negative control. We analyzed the intoxication level during the first 2 h after the injections, evaluating any recovery after 20 h after the injection as described by Valdez-Cruz et al. [51] and Estrada-Gomez et al. [52]. The intoxication levels were called ‘non-toxic’ when the animals showed no symptoms of envenoming within 20 h after testing, or showed the same symptoms as the control mice injected with 100 μL of saline.

### 4.4. Data Analysis

Probit LD_50_ calculations were performed in Microsoft Excel. All further statistical analyses were performed in R 3.3.1 (R Foundation, Vienna, Austria) [53]. Our own LD_50_ dataset was augmented with LD_50_ data from mice available in the literature. For some species, several LD_50_ values are available in the literature. When this occurred, we used the mean of the published values for further analysis. As the LD_50_ values span several orders of magnitude, they were log10 transformed prior to further statistical analysis. 

In order to see if closely related target species respond similarly, and thus can be used as generic models for the effects of scorpion venom in a large group of animals, Pearson’s and Spearman’s rank correlations were performed on the LD_50_ values of the scorpion venoms for each pair of target species. Conversely, to test if scorpion species were similar in their effects on the panel of target organisms, we performed correlations on the responses of the target organisms for the 10 scorpion species. 

To test if closely related scorpion species had a similar pattern of responses in the target organisms, we calculated the phylogenetic signal using Blomberg’s *K* [54]. For the scorpion dataset, CO1 sequences were used to calculate the phylogeny and branch lengths (See Table S1 in Supplementary Materials). The tree was calculated in MEGA 6.06 [55] using Maximum Likelihood under the GTR+G model, which was the best fitting model under the Bayesian information criterion (See Figure S1 in Supplementary Materials). We then used the mean path lengths method to estimate relative node age [56] and obtained an ultrametric phylogeny, setting the root age to one. A phylogeny with branch lengths of the target species was created from the Time Tree website (http://timetreebeta.igem.temple.edu/, see Figure S2 in Supplementary Materials) [57,58]. This tree was also scaled to a root age of one. Phylogenetic signal of the LD_50_ values was calculated using the function ‘physignal’ of the geomorph *R*-package [59], using 1000 replicates. Differences between buthids and non-buthids were tested per target species using a *t*-test. 

## Figures and Tables

**Table 1:** LD_50_ values for ten scorpion species in five target organisms. Values of LD_50_ values from experiments on mice, taken from the literature. In case several values were encountered in the literature, the mean was taken.

Family	Scorpion Species	LD50 (µg/mg Bodyweight)				*Mus* (mg/kg)	*Mus*	*Mus*
		*Tenebrio*	*Galleria*	*Gallus*	*Danio*	*Mean*	Lower	Higher
Buthidae	*Androctonus australis*	0.55	0.13	0.01	0.89	3.16	0.32 ^1^	6 ^2,3^
Buthidae	*Leiurus quinquestriatus*	0.18	0.17	0.0017	2.94	0.29	0.25 ^1,7^	0.33 ^2,3^
Buthidae	*Babycurus jacksoni*	3.17	0.53	0.0034	3.93			
Buthidae	*Buthus ibericus*	1.66	0.67	0.0007	12.1	1.17	0.9 ^8,^*	1.44 ^2,^*
Buthidae	*Centruroides gracilis*	1.53	1.25	0.014	4.25	2.7	2.7 ^9^	
Buthidae	*Grosphus grandidieri*	0.29	0.18	0.3057	3	13.13		
Caraboctonidae	*Hadrurus arizonensis*	1.3	0.63	0.0261	0.169	183	168 ^4^	198 ^5^
Iuridae	*Iurus dufoureius*	0.81	0.84	0.0029	0.0898	47.7	47.7 ^10^	
Scorpionidae	*Heterometrus laoticus*	1.64	0.4	0.0213	0.264	300	300 ^6,^*	
Scorpionidae	*Pandinus imperator*	1.4	0.29	0.0045	0.155	40	40 ^11,^*	

^1^ [33]; ^2^ [24]; ^3^ [34]; ^4^ [35]; ^5^ [36]; ^6^ [37]; ^7^ [38]; ^8^ [39]; ^9^ [40]; ^10^ [41]; ^11^ [42]. Values with an asterisk indicate values from closely related species.

**Table 2:** Phylogenetic signal (Blomberg’s *K*) of the LD_50_ across the scorpion species for each target organism. Significant *p*-values are in bold font.

Species	*K*	*p*
*Tenebrio*	0.56	0.607
*Galleria*	0.69	0.340
*Gallus*	0.76	0.335
*Danio*	1.25	0.031
*Mus*	1.48	0.011

**Table 3:** Correlation matrix. Pearson correlation coefficients of log10 transformed LD_50_ values above the diagonal, Pearson correlation coefficients of LD_50_ venom content below the diagonal.

Species	#	1	2	3	4	5	6	7	8	9
*Androctonus australis*	1		0.99 **	0.61	0.99 **	0.95 *	0.13	0.11	0.08	0.16
*Leiurus quinquestriatus*	2	0.99 **		0.49	1.00 **	0.99 *	−0.01	−0.04	−0.06	0.01
*Buthus ibericus*	3	0.61	0.49		0.51	0.34	0.86	0.85	0.84	0.88
*Centruroides gracilis*	4	0.99 **	1.00 **	0.51		0.98 *	0.01	−0.01	−0.04	0.04
*Grosphus grandidieri*	5	0.95 *	0.99 *	0.34	0.98 *		−0.18	−0.2	−0.23	−0.15
*Hadrurus arizonensis*	6	0.13	−0.01	0.86	0.01	−0.18		0.99 **	1.00 **	0.99 **
*Iurus dufoureius*	7	0.11	−0.04	0.85	−0.01	−0.2	0.99 **		1.00 **	1.00 **
*Heterometrus laoticus*	8	0.08	−0.06	0.84	−0.04	−0.23	1.00 **	1.00 **		1.00 **
*Pandinus imperator*	9	0.16	0.01	0.88	0.04	−0.15	0.99 **	1.00 **	1.00 **	

Values with a single asterisk were significant at an α of 0.05, values with two asterisks were significant after Holm’s correction for multiple comparisons.

**Table 4:** Correlation matrix. Pearson correlation coefficients of log10 transformed LD_50_ values above the diagonal, Spearman rank correlation coefficients below the diagonal. None of the correlations were significant after Holms correction for multiple comparisons. With the exception of the two insect species, no target organism positively correlates with another, showing that results in one system cannot be considered indicative for other target organisms.

Target species	*Tenebrio*	*Galleria*	*Danio*	*Gallus*	*Mus*
*Tenebrio*		0.7 *	−0.24	−0.17	0.49
*Galleria*	0.63		−0.07	−0.22	0.24
*Danio*	0.17	0.03		−0.1	−0.8 *
*Gallus*	−0.12	−0.08	−0.08		0.44
*Mus*	0.22	0.17	−0.72 *	0.57	

Values with an asterisk were significant at an α of 0.05.

## Supplementary Materials

The following are available online at www.mdpi.com/2072-6651/9/10/312/s1, Table S1: Table with GenBank CO1 accession numbers. Voucher numbers refer to the specimens in the collection of CIBIO/InBio at the University of Porto, Table S2: Mean dry venom compound per individual. Several milkings were made per (sub)adult specimen in some cases, Table S3: Toxicological analysis of the venom of Grosphus grandidieri. “Toxic” means that the mice showed symptoms such as: pain, piloerection, excitability, salivation, lacrimation, dyspnea, diarrhea, temporary paralysis, but recovered within 20 h. “Lethal” means that the mice showed some or all the symptoms of intoxication and died within 20 h after injection, Figure S1: Phylogeny of scorpions based on CO1 sequences. Note that Grosphus flavopiceus has been used to represent the position of G. grandidieri, and likewise a sequence of Iurus kraepelini has been used to represent I. dufoureius. The branch separating Buthus ibericus from the clade containing Androctonus australis and Leiurus quinquestriatus is very short, Figure S2: Time tree of target organisms used to calculate phylogenetic signal, built using the online service TimeTree (http://timetreebeta.igem.temple.edu/). Numbers at branches indicate divergence times in millions of years.
