# Supplementary material for: Target-Specificity in Scorpions; Comparing Lethality of Scorpion Venoms across Arthropods and Vertebrates
Source: Toxins (Basel). 2017 Oct 4;9(10):312. doi: 10.3390/toxins9100312 (PMC5666359; doi:10.3390/toxins9100312)
Supplement: Supplementary file 1 [file toxins-09-00312-s001.pdf]

Supplementary Materials: Target-specificity in scorpions; Comparing lethality of scorpion venoms across arthropods and vertebrates

Arie van der Meijden, Bjørn Koch, Tom van der Valk, Leidy J. Vargas-Muñoz and Sebastian Estrada-Gómez

Table S1. Table with GenBank CO1 accession numbers. Voucher numbers refer to the specimens in the collection of CIBIO/InBio at the University of Porto.

Species	Voucher	Accession number
Androctonus australis	Sc904	gi379647585
Leiurus quinquestriatus	Sc1062	gi379647601
Buthus ibericus	Sc112	gi290918312
Grosphus flavopiceus	Sc1085	gi379647593
Centruroides gracilis		gi71743793
Heterometrus laoticus	Sc1084	gi555299473
Pandinus imperator	Sc1050	gi379647587
Hadrurus arizonensis	Sc1042	gi379647597
Iurus kraepelini	Sc866	gi379647589

Table S2. Mean dry venom compound per individual. Several milkings were made per (sub)adult specimen in some cases.

Species	*n*	Dry venom (mg)	mg/milking
Androctonus australis	21	23.5	1.12
Leiurus quinquestriatus	51	25.1	0.49
Buthus ibericus	47	41.6	0.89
Centruroides gracilis	42	22.5	0.54
Babycurus jacksoni	38	53.9	1.42
Grosphus grandidieri	19	103.9	5.47
Hadrurus arizonensis	9	75.3	8.37
Iurus sp.*	12	35.2	2.93
Heterometrus laoticus	21	119	5.67
Pandinus imperator	15	72.7	4.85

*2 I. dufoureius, 6 I. kraepelini, 4 I. sp.

Table S3. Toxicological analysis of the venom of *Grosphus grandidieri*. "Toxic" means that the mice showed symptoms such as: pain, piloerection, excitability, salivation, lacrimation, dyspnea, diarrhea, temporary paralysis, but recovered within 20 h. "Lethal" means that the mice showed some or all the symptoms of intoxication and died within 20 h after injection.

**Time mins**	**10 mins**	**15 mins**	**20 mins**	**30 mins**	**40 mins**	**50 mins**	**>60 mins**	
**Group 1**<br>**Non-lethal**<br>**100μg/animal**	None	None	None	Lacrimation	Lacrimation<br>Erratic movements<br>Pain	Lacrimation<br>Erratic movements<br>Pain	Lacrimation<br>Erratic movements<br>Pain<br>Diahrrea	Recovered after 24 h
**Group 2**<br>**Non-lethal**<br>**150 μg/animal**	None	None	Salivation	Lacrimation<br>Pain	Lacrimation<br>Erratic movements<br>Pain	Lacrimation<br>Erratic movements<br>Pain	Lacrimation<br>Erratic movements<br>Pain<br>Diahrrea	Recovered after 24 h
**Group 3**<br>**Toxic**<br>**Lethal**<br>**250 μg/animal**	None	None	Erratic movements	Pain<br>Lacrimation<br>Erratic movements<br>Salivation	Pain<br>Lacrimation<br>Erratic movements<br>Salivation	Pain<br>Lacrimation<br>Erratic movements<br>Salivation	Pain<br>Lacrimation<br>Erratic movements<br>Salivation	
**Group 4**<br>**Toxic**<br>**Lethal**<br>**350 μg/animal**			Pain<br>Erratic movements	Pain<br>Lacrimation<br>Erratic movements<br>Salivation	Pain<br>Lacrimation<br>Erratic movements<br>Salivation	Pain<br>Lacrimation<br>Erratic movements<br>Salivation	Pain<br>Lacrimation<br>Erratic movements<br>Salivation	
**Group 5**<br>**Toxic**<br>**Lethal**<br>**400 μg/animal**	None	Pain	Pain<br>Lacrimation<br>Erratic movements	Pain<br>Lacrimation<br>Erratic movements<br>Salivation	Pain<br>Lacrimation<br>Erratic movements<br>Salivation	Pain<br>Lacrimation<br>Erratic movements<br>Salivation<br>Temporary paralysis<br>Diahrrea	Death	
**Group 6**<br>**Lethal**<br>**1000 μg/animal**	Pain	Pain<br>Salivation<br>Lacrimation	Pain<br>Salivation<br>Lacrimation<br>Erratic movements	Death				

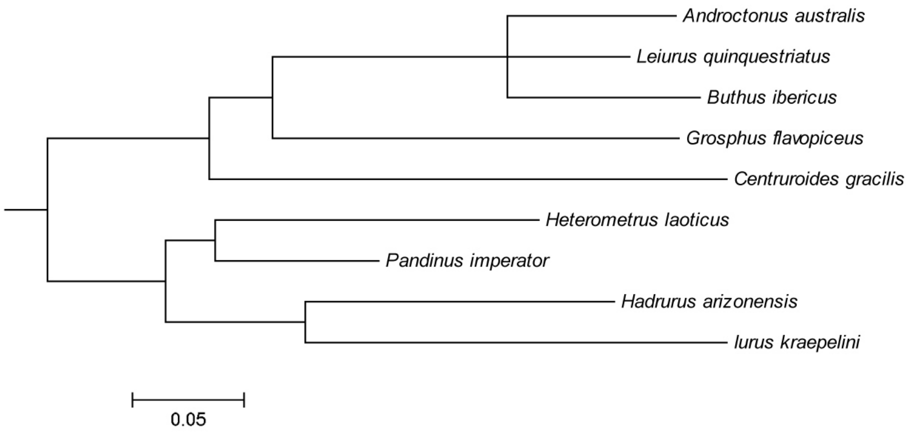

Figure S1. Phylogeny of scorpions based on CO1 sequences. Note that *Grosphus flavopiceus* has been used to represent the position of *G. grandidieri*, and likewise a sequence of *Iurus kraepelini* has been used to represent I. dufoureius. The branch separating *Buthus ibericus* from the clade containing *Androctonus australis* and *Leiurus quinquestriatus* is very short.

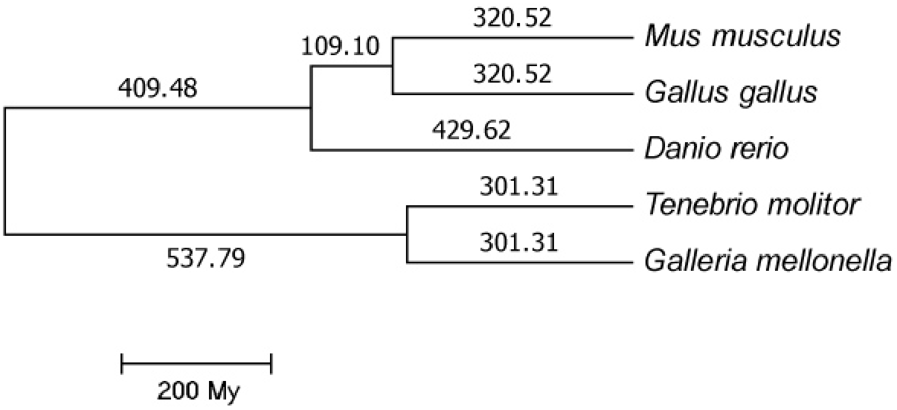

Figure S2. Time tree of target organisms used to calculate phylogenetic signal, built using the online service TimeTree (http://timetreebeta.igem.temple.edu/). Numbers at branches indicate divergence times in millions of years.

© 2017 by the authors; licensee MDPI, Basel, Switzerland. This article is an open access article distributed under the terms and conditions of the Creative Commons by Attribution (CC-BY) license (http://creativecommons.org/licenses/by/4.0/).
